# PGC1α Plays a Critical Role in TWEAK–Induced Cardiac Dysfunction

**DOI:** 10.1371/journal.pone.0054054

**Published:** 2013-01-16

**Authors:** Jianru Shi, Bingbing Jiang, Yiling Qiu, Jian Guan, Mohit Jain, Xin Cao, Michael Bauer, Lihe Su, Linda C. Burkly, Teresa C. Leone, Daniel P. Kelly, Ronglih Liao

**Affiliations:** 1 Cardiac Muscle Research Laboratory, Cardiovascular Division, Brigham and Women’s Hospital, Harvard Medical School, Boston, Massachusetts, United States of America; 2 Protein Biochemistry Department, Biogen Idec, Inc, Cambridge, Massachusetts, United States of America; 3 Immunology Department, Biogen Idec, Inc, Cambridge, Massachusetts, United States of America; 4 Cardiovascular pathobiology program, Sanford-Burnham Medical Research Institute at Lake Nona, Orlando, Florida, United States of America; Brigham and Women’s Hospital, United States of America

## Abstract

**Background:**

Inflammatory cytokines play an important role in the pathogenesis of heart failure. We have recently found the cytokine TWEAK (tumor necrosis factor (TNF)-like weak inducer of apoptosis), a member of the TNF superfamily, to be increased in patients with cardiomyopathy and result in the development of heart failure when overexpressed in mice. The molecular mechanisms underlying TWEAK-induced cardiac pathology, however, remain unknown.

**Methodology and Critical Finding:**

Using mouse models of elevated circulating TWEAK levels, established through intravenous injection of adenovirus expressing TWEAK or recombinant TWEAK protein, we find that TWEAK induces a progressive dilated cardiomyopathy with impaired contractile function in mice. Moreover, TWEAK treatment is associated with decreased expression of peroxisome proliferator-activated receptor gamma coactivator-1α (PGC1α) and genes required for mitochondrial oxidative phosphorylation, which precede the onset of cardiac dysfunction. TWEAK-induced downregulation of PGC1α requires expression of its cell surface receptor, fibroblast growth factor-inducible 14 (Fn14). We further find that TWEAK downregulates PGC1α gene expression via the TNF receptor-associated factor 2 (TRAF2) and NFκB signaling pathways. Maintaining PGC1α levels through adenoviral-mediated gene expression is sufficient to protect against TWEAK-induced cardiomyocyte dysfunction.

**Conclusion:**

Collectively, our data suggest that TWEAK induces cardiac dysfunction via downregulation of PGC1α, through FN14-TRAF2-NFκB-dependent signaling. Selective targeting of the FN14-TRAF2-NFκB-dependent signaling pathway or augmenting PGC1α levels may serve as novel therapeutic strategies for cardiomyopathy and heart failure.

## Introduction

Tumor necrosis factor (TNF)-like weak inducer of apoptosis (TWEAK) is a recently identified member of the TNF superfamily cytokines [1]. Endogenously, TWEAK initially exists as a 249-amino acid type II transmembrane homotrimer that is processed rapidly into a soluble, circulating cytokine containing 156 amino acids [2]. TWEAK is a multifunctional cytokine involved in regulating many biological processes including cell proliferation, differentiation, apoptosis, migration, angiogenesis, and inflammation through binding to its cell surface signaling receptor, FGF-inducible molecule 14 (Fn14) [3], [4], [5]. TWEAK and Fn14 are typically expressed at relatively low levels in healthy conditions, and their upregulation is linked with deleterious pathologic conditions and disease states, such as renal damage, hypoxia/reoxygenation, inflammatory diseases, and muscle dysfunction [1], [6], [7], [8]. Recently, we have identified an essential role for the TWEAK-Fn14 axis in the development of dilated cardiomyopathy [9]. Circulating levels of TWEAK were found to be elevated in patients with idiopathic dilated cardiomyopathy and overexpression of TWEAK resulted in structural remodeling and heart failure in mice [9]. Consistent with our findings, TWEAK levels have also been found to be increased acutely in patients with myocardial infarction and may predict short-term adverse events [10].

In the present study, we find that TWEAK-induced cardiac pathology in mice is associated with mitochondrial dysfunction. We further identify peroxisome proliferator-activated receptor gamma coactivator 1α (PGC1α), an essential regulator of mitochondrial biogenesis and energy metabolism [11], [12], to be downregulated in hearts from mice with increased circulating TWEAK levels and in isolated cardiomyocytes exposed to TWEAK. TWEAK-mediated downregulation of PGC1α, is found to occur via an FN14-TRAF2-NFκB-dependent signaling pathway and maintenance of PGC1α levels are shown to protect against TWEAK-induced cardiac dysfunction. Selective targeting of the FN14-TRAF2-NFκB-dependent signaling pathway or augmenting PGC1α levels may serve as novel therapeutic strategies for cardiomyopathy and heart failure.

## Materials and Methods

### Animals

C57BL/6J male mice (8 weeks old) were purchased from Jackson Laboratory. Fn14 knockout mice (FN14 KO) and wild-type (WT) counterparts have been previously reported and were generated on the 129 strain background and backcrossed onto the C57BL/6 strain, as described [13], [14]. To study the *in vivo* role of TWEAK, adenovirus expressing soluble murine TWEAK (Ad-TWEAK) or control adenovirus expressing green fluorescent protein (Ad-GFP) was delivered intravenously to mice through tail vein injection at a dose of 10^11^ viral particles per mouse as described previously [9]. In separate animals, recombinant Fc-TWEAK (rTWEAK), a fusion protein of TWEAK with the murine IgG2a Fc region, or an isotype-matched control IgG at a dose of 200 µg per mouse was injected into mice intravenously via tail vein injection. All animal procedures and handling were carried out with the approval of Harvard Medical School, the Institutional Animal Care and Use Committee (IACUC).

### Non-invasive Transthoracic Echocardiography

Longitudinal cardiac function and chamber structure was assessed in conscious mice through serial non-invasive transthoracic echocardiography, using a Vevo2100 system (VisualSonics) as previously described [15].

### Cardiomyocyte Isolation and Culture

Adult rat ventricular cardiomyocytes were isolated from male Wistar rats (Charles River Laboratories) using collagenase perfusion and cell dissociation, as described previously [16]. Isolated cardiomyocytes were cultured in DMEM overnight prior to incubation with 100 ng/ml of rTWEAK or control IgG at the designated time. Similarly, adult ventricular cardiomyocytes were isolated from WT and Fn14 KO mice using our established protocols [17], and treated with IgG or rTWEAK as described above.

### Cell Contractility Measurement

Adult rat cardiomyocytes were infected with adenovirus expressing PGC1α (Ad-PGC1α) or Ad-GFP [18] for 24 hours prior to treatment with 100 ng/ml IgG or rTWEAK for 48 hours. Cardiomyocytes were then perfused with 1.2 mM Ca^2+^ Tyrode’s buffer at 37°C and field stimulated at a frequency of 5 Hz. Cell shortening/relengthening was measured using video edge detection (IonOptix, Milton, MA), as we have previously described [16]. Percent cell shortening (%CS) was calculated as the ratio of the shortening length during systole over diastolic cell length. Time to 90% relaxation defined as the time required from the peak contraction to 90% relaxation.

### TMRE (tetramethylrhodamine ethyl ester) Staining

Cardiomyocytes were cultured in petri-dishes (35 mm×15 mm) or 24-well plates overnight and subsequently treated with 100 ng/ml IgG or rTWEAK for 24 hours, followed by incubation with 10 nM TMRE for 30 minutes. After washing with pre-warmed PBS, culture dishes were placed in a LSM700 confocal microscopy equipped with temperature-controlled chamber for live cell imaging. TMRE fluorescence was assessed by excitation at 555 nm. On average, 5–7 pictures were taken from each dish. Cardiomyocytes were hand-traced and quantified using SigmaPro software.

### Membrane Protein Isolation

Membrane protein was isolated using a subcellular protein fractionation kit (Thermo Scientific). Cardiomyocytes were detached from culture dishes using a cell scraper and harvested into ice-cold PBS. Cells were centrifuged at 500 ×g for 5 minutes at 4°C, washed with ice-cold PBS, and centrifuged again at 500 ×g for 2 minutes. The cell pellet was suspended in cytoplasmic extraction buffer containing protease inhibitors and incubated at 4°C for 10 minutes with gentle mixing. After centrifugation at 500 ×g for 5 minutes, the separated supernatant contained the cytoplasmic fraction. The remaining pellet was resuspended in membrane extraction buffer containing protease inhibitors, incubated at 4°C for 10 minutes with gentle mixing, and then centrifuged at 3,000 ×g for 5 minutes. The resulting supernatant fraction provided the membrane proteins.

### RNA Isolation and qRT-PCR

RNA was extracted using Trizol reagent (Invitrogen). Genomic DNA was removed by using Turbo-DNA free kit (Ambion). iScriptTM cDNA Synthesis Kit (Bio-Rad) was used for cDNA synthesis and quantitative RT-PCR was performed using a CFX96 real-time PCR system (Bio-Rad). Primers used for qPCR include: PGC1α forward GACCCTCCTCACACCAAAC, reverse GCGACTGCGGTTGTGTATG; GAPDH forward GGTGATGCTGGTGCTGAGTA, reverse TTGCTGACAATCTTGAGGGA; Cyt C forward AGGCTGCTGGATTCTCTTAC, reverse ATTAGGTCTGCCCTTTCTCC; Cox2 forward ATTGCTCTCCCCTCTCTAC, reverse GGTTTTAGGTCGTTTGTTGG; Atp5o forward ATGCAACCGCCCTGTACTC, reverse GAACAGCCAGAGACACTTTG; Ndufb5 forward ACTATGACGCTCGCTTCTTG, reverse TAGCCTTCTGGGATTTCTGC; and Sdhα forward CTGCATTTGGCCTTTCTGAG, reverse GTTGTCCTCTTCCATGTTCC.

### Western Blot Analysis

For protein isolation, heart tissues were homogenized or cultured cardiomyocytes were harvested in cell lysis buffer (Cell Signaling). Equal amounts of proteins were used for SDS/PAGE and electrotransferred to a PVDF membrane (Millipore). The membranes were treated with Odyssey Blocking buffer (Li-Cor) for 1 hour and incubated with appropriate primary antibodies overnight at 4°C. After washing, blots were incubated with corresponding secondary antibodies conjugated with IRDye 800CW or IRDye 680LT. Blots were then scanned and analyzed using the Odyssey infrared scanner (Li-Cor).

### Materials

Low glucose DMEM and Laminin were purchased from Invitrogen. SC-514 and antibody against PGC1α were obtained from Calbiochem. Antibodies against p-p65, p-IκBα and IκBα were obtained from Cell Signaling Technology. Antibody against GAPDH was obtained from R&D Systems. Ad-GFP and Ad-TWEAK were provided by Biogen Idec Inc.

### Statistical Analysis

All data are expressed as mean ± SEM. Student’s *t* test and ANOVA analyses were used for statistical comparison between two groups and multiple groups, respectively. A *P* value <0.05 was considered to be significant.

## Results

### TWEAK Induces Cardiomyopathy with Mitochondrial Damage and Decreased Expression of PGC1α

**Figure 1:**
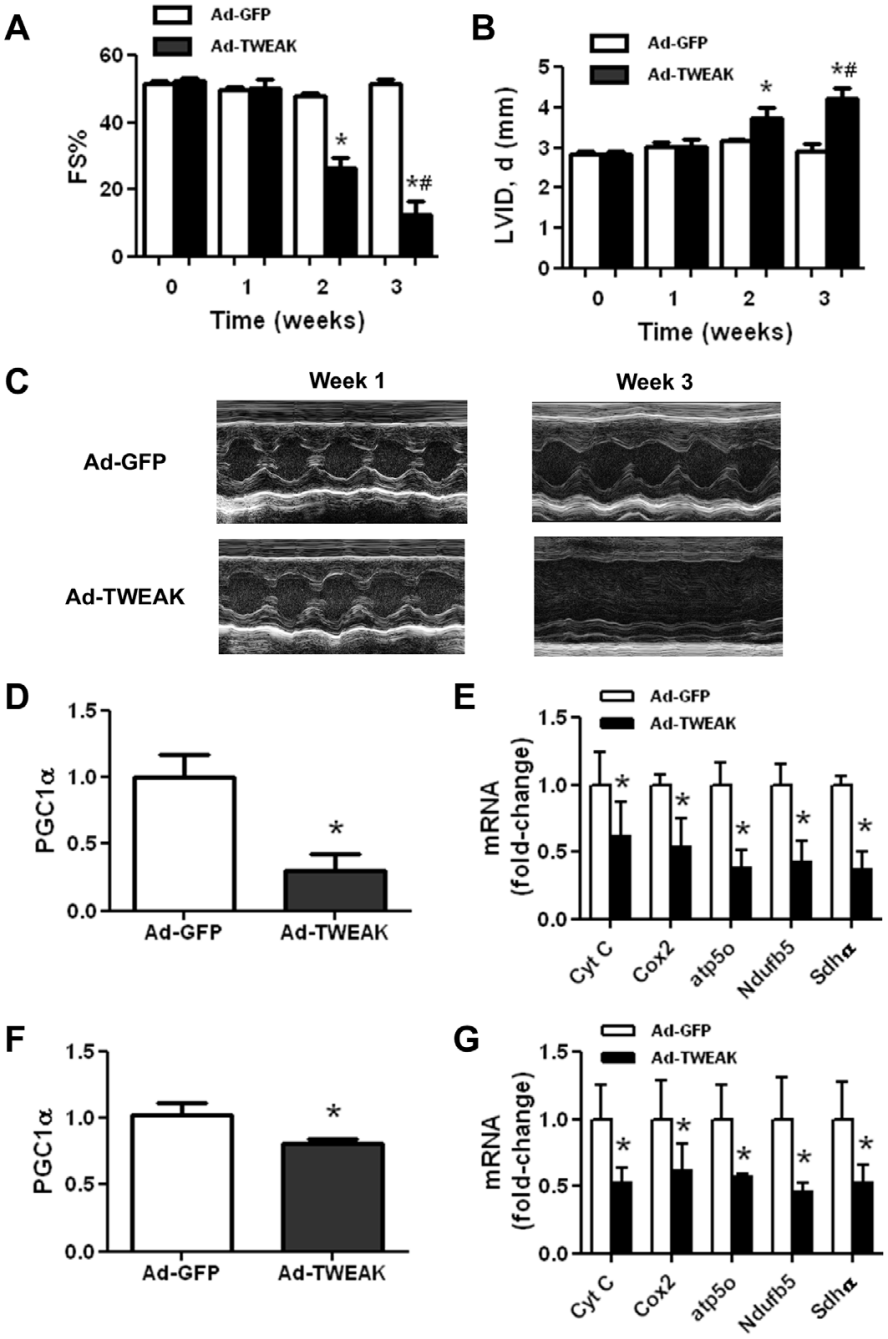
TWEAK induces cardiomyopathy and metabolic reprogramming in mouse hearts. (A) Fractional shortening (FS%, calculated as the difference in chamber dimension between diastole and systole over the chamber dimension in diastole) and (B) LV ventricular diastolic chamber dimension (LVID) were determined in mice by transthoracic echocardiography 1-day prior as well as 1, 2, and 3-week after intravenously delivery of control, Ad-GFP or Ad-TWEAK. (C) Representative M-Mode echocardiographic images at one week and three weeks post Ad-GFP and Ad-TWEAK injection in mice. Real time PCR analysis for expression of (D) PGClα and (E) OXPHOS genes in cardiac samples at 3-weeks post-Ad-GFP or Ad-TWEAK injection. Real time PCR analysis for (F) PGClα and (G) OXPHOS genes in cardiac samples at 1 week (prior to the development of heart failure) post-Ad-GFP or Ad-TWEAK injection. All real time PCR data were normalized to β-actin and presented relative to the Ad-GFP group. * p<0.05 vs. Ad-GFP, # p<0.05 vs. Ad-TWEAK-injected mice at 1 week time point, N = 5 for each group.

To elucidate the underlying mechanisms by which TWEAK induces cardiac dysfunction, we utilized our established mouse model of TWEAK induced cardiomyopathy. Increased circulating level of TWEAK was achieved via adenovirus-mediated gene delivery as we described previously [9]. Consistent with our previous observations, mice receiving Ad-TWEAK developed a gradual decrease in contractile function and enlarged ventricular chamber dimensions *in vivo,* measured by serial non-invasive transthoracic echocardiography (Figures 1A–1C). Derangements in myocardial substrate utilization have been shown to result in cardiac dysfunction [19], [20], [21]. We, therefore, measured the level of PGC1α, a transcriptional co-activator that is essential for mitochondrial biogenesis and expression of genes involved in cardiac metabolism, including oxidative phosphorylation (OXPHOS) genes such as Cyt C, Cox2, Atp5o, Ndufb5 and Sdhα. PGC1α expression was significantly downregulated in Ad-TWEAK-injected mice in which cardiac dysfunction was readily apparent (3 week time-point) (Figure 1D), and in agreement with the down-regulation of PGC1α, cardiac expression of multiple OXPHOS genes was also significantly decreased (Figures 1E). Importantly, we found that the downregulation of PGC1α and OXPHOS gene expression occurred as early as 1-week after Ad-TWEAK injection (Figures 1F and 1G), which preceded the development of cardiac dysfunction. These results suggest that the decreased PGC1α expression may play a causal role in mediating TWEAK-induced cardiac dysfunction.

**Figure 2:**
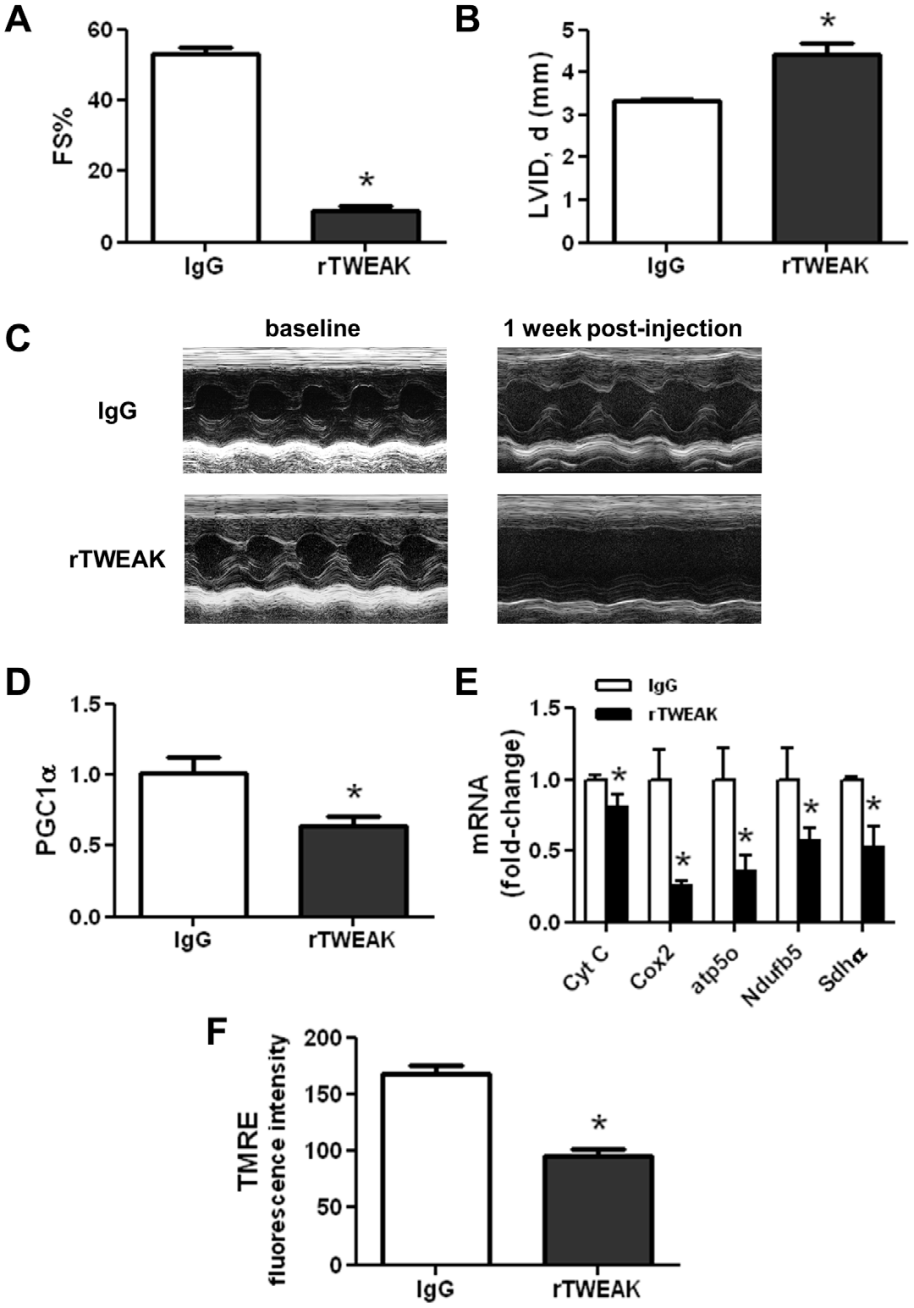
rTWEAK delivery results in cardiomyopathy and altered metabolic gene expression. (A) Fractional shortening (FS%) and (B) Left ventricular diastolic chamber dimension (LVID), as determined by transthoracic echocardiography in mice one week after intravenous delivery of IgG or rTWEAK. (C) Representative M-Mode echocardiographic images before and following IgG or rTWEAK injection in mice. Real time PCR analysis of heart samples for expression of (D) PGClα and (E) OXPHOS genes, normalized to β-actin and presented relative to the IgG group. (F) Mitochondria membrane potential in IgG and rTWEAK treated cardiomyocytes. The membrane potential was measured by TMRE staining. The fluorescent intensities of TMRE were normalized to the total number of cardiomyocytes in the respective fields. * p<0.05 vs. IgG, N = 3 for each group.

To exclude the potential effects of adenoviral delivery and gene expression in the Ad-TWEAK mouse model, we developed a novel mouse model with tail vein delivery of recombinant TWEAK (rTWEAK). As shown in Figures 2A–2C, delivery of rTWEAK resulted in the development of cardiac dysfunction with a significant decrease in contractile function and ventricular dilation after 1-week of delivery. This degree of dysfunction is comparable to that in Ad-TWEAK-injected mice at 3-weeks. Furthermore, similar to Ad-TWEAK-injected mice, rTWEAK administration significantly downregulated the cardiac expression of PGC1α and multiple OXPHOS genes (Figures 2D and 2E). Using TMRE, a mitochondrial membrane potential sensitive fluorescence dye, we observe a significantly reduction in TMRE fluorescence signal in cardiomyocytes exposed to rTWEAK as compared to IgG (Figure 2F), indicating that mitochondrial membrane potential was reduced by direct exposure of cells to TWEAK.

### Fn14 is Necessary for TWEAK-mediated Changes in PGC1α Expression and TRAF2 Translocation

**Figure 3:**
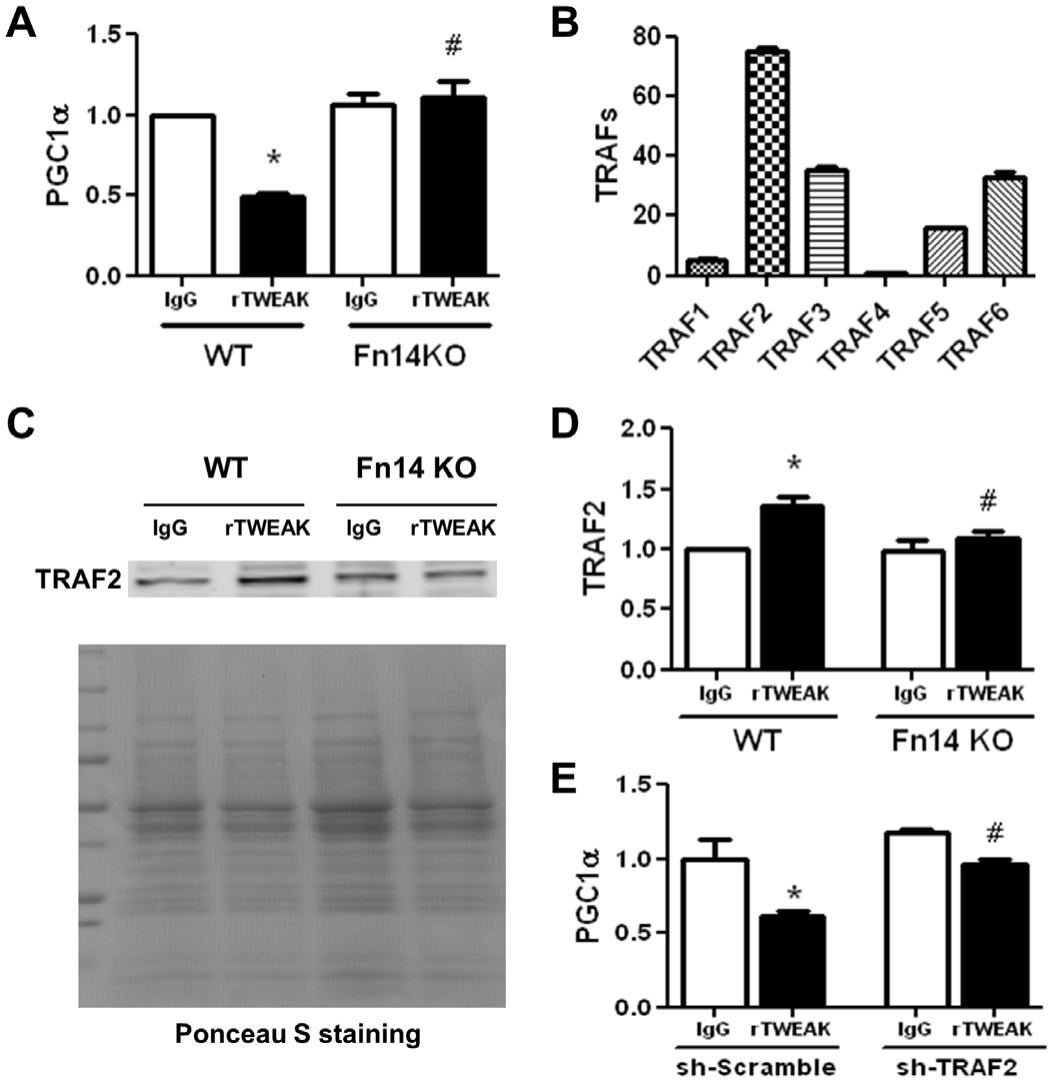
Fn14 is required for TWEAK-mediated down-regulation of PGC1α expression and TRAF2 membrane translocation. Cardiomyocytes isolated from WT and Fn14 KO mice were treated with 100 ng/ml IgG or rTWEAK, and then harvested for determination of PGC1α expression and TRAF2 membrane translocation. (A) Real time PCR analysis for PGClα expression post IgG and rTWEAK treatment, normalized to β-actin and presented relative to the IgG treated WT group. (B) Real time PCR analysis for gene expression profiling of TRAF family proteins, expressed as the mRNA ratio of TRAF/β-actin. (C) Immunoblot analysis for TRAF2 membrane translocation after IgG and rTWEAK treatment for 10 min. Lower panel shows Ponceau S staining of PVDF membranes that was used for evaluation of protein loading amount. (D) Bar-graph shows the relative levels of TRAF2 by densitomitric analysis with IgG treated WT group as 1-fold. * p<0.05 vs. IgG treatment in WT group, # p<0.05 vs. rTWEAK treatment in WT group. (E) Silencing of TRAF2 using shRNA (sh-TRAF2) prevents TWEAK-induced downregulation of PGC1α. * p<0.05 vs. IgG in sh-Scramble group, and # p<0.05 vs. rTWEAK in sh-Scramble group.

We have previously identified Fn14 as a critical receptor that mediates TWEAK-induced cardiac dilatation and dysfunction [9]. To determine whether Fn14 is also necessary for TWEAK-induced downregulation of PGC1α expression, PGC1α gene expression in response to TWEAK treatment was examined in cultured cardiomyocytes isolated from adult WT and Fn14 knockout mice. Loss of Fn14 completely abolished TWEAK-mediated downregulation of PGC1α (Figure 3A).

Activation of Fn14 may result in membrane translocation of TRAF (tumor necrosis factor receptor associated factor) leading to activation of downstream signaling pathways [22]. Gene expression profiling of TRAF family proteins revealed that among all six TRAF family genes examined, TRAF2 is the most abundant in adult cardiomyocytes (Figure 3B). Subcellular fractionation of cardiomyocytes after rTWEAK or IgG treatment for 10 minutes showed that TWEAK increased membrane translocation of TRAF2 proteins, in an Fn14 dependent manner (Figure 3C and 3D). Importantly, silencing TRAF2 expression by shRNA targeting TRAF2 (sh-TRAF2) in cardiomyocytes partially reversed PGC1α expression following TWEAK treatment, while scramble shRNA (sh-Scramble) showed no effect (Figure 3E).

### Activation of NFκB Mediates TWEAK-induced Downregulation of PGC1α Expression

**Figure 4:**
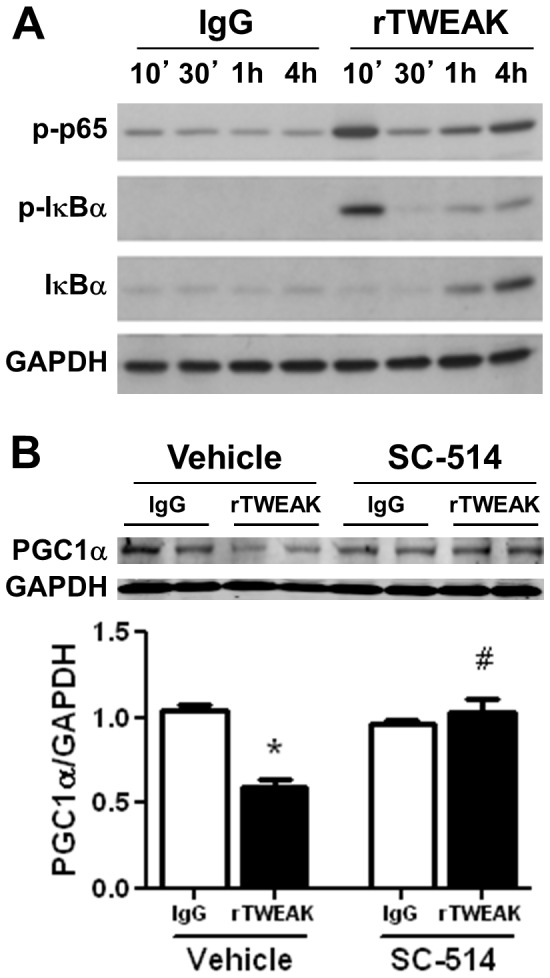
Activation of NFκB mediates TWEAK-induced downregulation of PGC1α. (A) Immunoblots of phospho-p65, phospho-IκBα, total-IκBα and GAPDH in isolated cardiomyocytes incubated with 100 ng/ml IgG or rTWEAK at designated time points. (B) Inhibition of NFκB activation with SC-514 (25µM) abolished TWEAK-mediated downregulation of PGC1α expression. * p<0.05 vs. IgG and # p<0.05 vs. rTWEAK in the absence of SC-514.

Treatment of cardiomyocytes with rTWEAK, but not IgG, further induced downstream mediators, including IκBα phosphorylation, degradation, and resynthesis, accompanied by the phosphorylation of NFκB p65, which occurred as early as 10 minutes and persisted for several hours (Figure 4A). As shown in Figure 4B, inhibition of NFκB activation via a selective IκB kinase-β inhibitor, SC-514, completely abolished TWEAK regulation of PGC1α expression, indicating a requirement for NFκB activation in the downregulation of PGC1α.

### Maintaining PGC1α Prevents TWEAK-induced Cardiomyocyte Contractile Dysfunction

**Figure 5:**
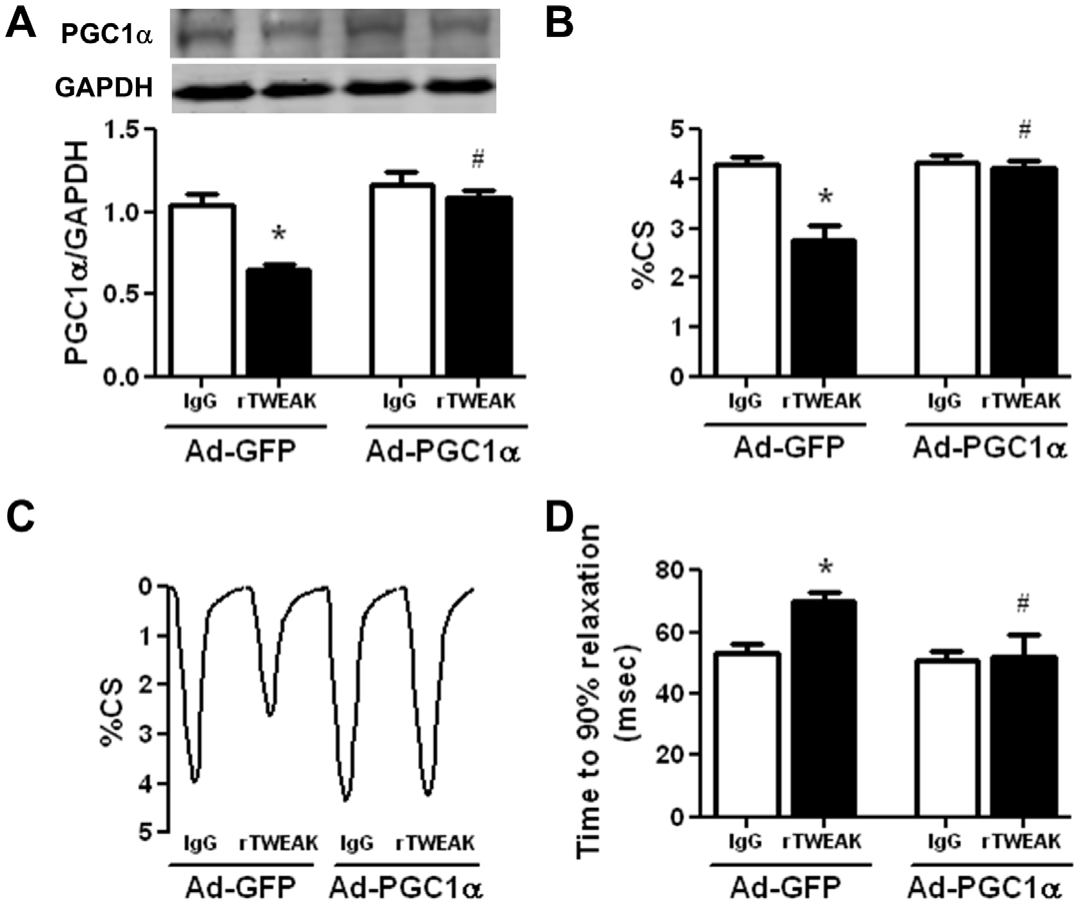
Maintenance of PGC1α levels protects against TWEAK-induced contractile dysfunction in isolated cardiomyocytes. Isolated cardiomyocytes were infected with adenovirus (MOI of 10) expressing GFP or PGC1α for 24 hours prior to treatment with IgG or rTWEAK for 48 hours. (A) Western blot analysis of PGC1α expression, normalized with GAPDH. N = 3 independent biological replicates group. (B) Isolated cardiomyocyte function (%CS) was determined using edge detection method. (C) Representative tracings of single cell shortening at indicated conditions. (D) Time to 90% relaxation in isolated cardiomyocytes. Cellular function was assessed in three independent biological replicates and data from 8–12 cells was averaged as N = 1 for a given experiment. * p<0.05 vs. IgG and # p<0.05 vs. rTWEAK in Ad-GFP group.

To test our hypothesis that the downregulation of PGC1α plays a causal role in TWEAK-induced cardiac dysfunction, adenoviral-mediated PGC1α expression was used in cultured cardiomyocytes for 24 hours prior to treatment with IgG or rTWEAK. Upon rTWEAK treatment, PGC1α expression was downregulated in Ad-GFP-infected cells but maintained at normal levels in Ad-PGC1α-infected cells (Figure 5A). rTWEAK significantly impaired cardiomyocyte contractility as revealed by reduced percent cell shortening (%CS) (Figures 5B and 5C) and prolonged time of cell relaxation (Figure 5D). Strikingly, maintaining PGC1α expression prevented rTWEAK impaired cell contractility (Figures 5B–5D). These data support the notion that downregulation of PGC1α plays a critical role in mediating TWEAK-induced cardiomyocyte dysfunction.

## Discussion

**Figure 6:**
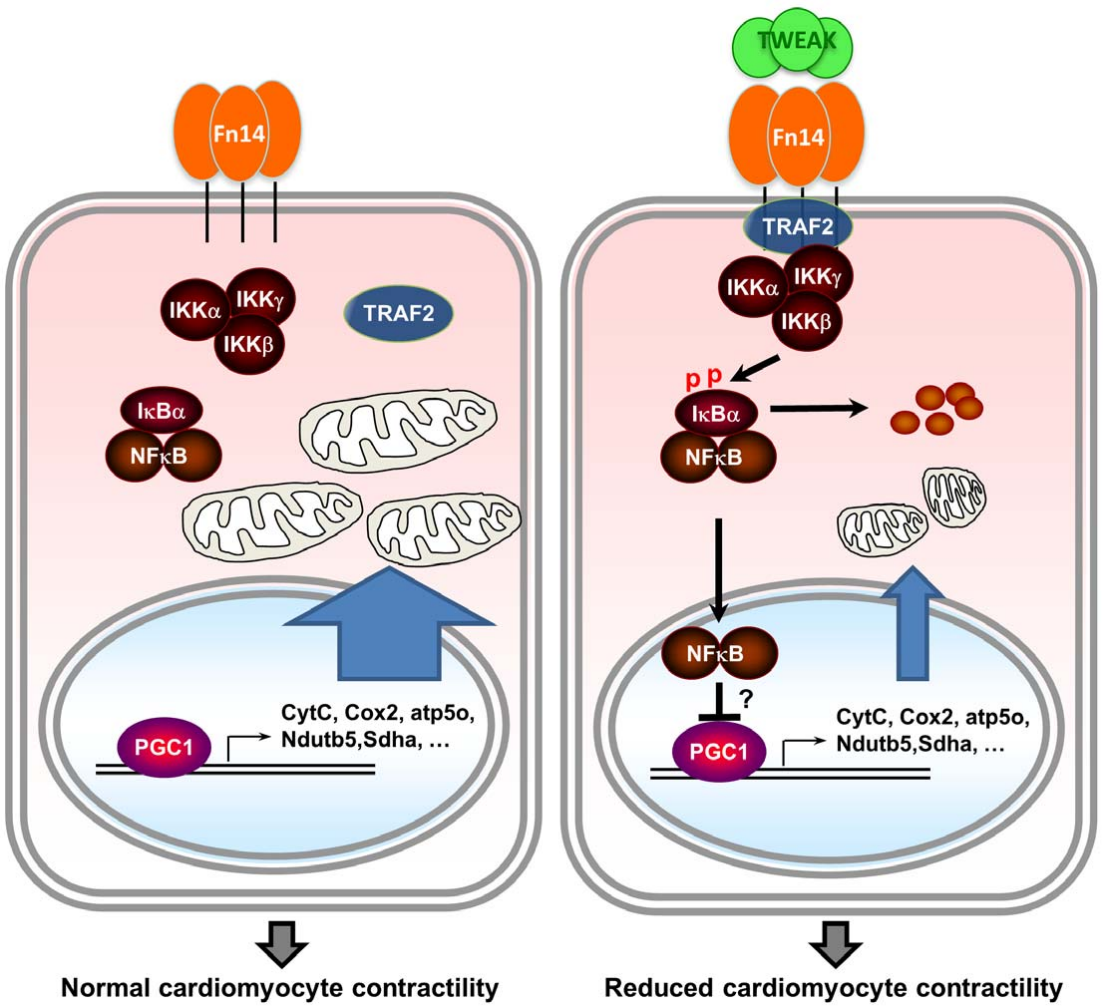
Graphical illustration summarizing the proposed mechanism underlying TWEAK-induced cardiac dysfunction. Normal PGC1 levels maintain OXPHOS gene expression and mitochodrial function to support cardiomyocyte contractility (left panel). TWEAK downregulates PGC1 levels through FN14-TRAF2-NFκB-dependent signaling, leading to impaired OXPHOS gene expression and mitochondrial dysfunction, and reduced cardiomyocyte contractility (right panel).

In the present study, we reveal that the cytokine TWEAK downregulates PGC1α and mitochondrial OXPHOS gene expression in cardiomyocytes, which contributes to TWEAK-induced cardiac dysfunction. Moreover, we find that TWEAK regulates PGC1α expression via Fn14/TRAF2/NFκB-dependent signaling pathways (Figure 6).

PGC1α is a transcriptional coactivator that is preferentially expressed in tissues with high-energy demand and greater mitochondrial abundance, including the heart [23], [24]. PGC1α directly coactivates the transcription factors PPARs and estrogen-related receptors (ERRs) and regulates mitochondrial fatty acid β-oxidation as well as expression of genes related to electron transport chain and oxidative phosphorylation [24]. PGC1α also coactivates nuclear respiratory factor (NRF) and the expression of mitochondrial transcription factor A (Tfam), which are essential for mitochondrial DNA replication and transcription [25]. Expression levels of PGC1α are intricately linked to the maintenance of the cardiac structure and function. Genetic ablation or overexpression of PGC1α has been demonstrated to result in cardiac dysfunction [18], [26], [27], thereby suggesting that PGC1α is required for maintenance of proper heart function. Interestingly, we observed a reduced expression of PGC1α and OXPHOS genes in response to TWEAK treatment. Importantly, the downregulation of these genes occurred while heart function was still maintained and prior to the development of functional decline, suggesting a temporal association between suppression of PGC1α expression by TWEAK and the development of cardiac dysfunction. In contrast, we have previously found that genetic overexpression of TWEAK does not result in increased cardiac apoptosis, unlike with other cytokines, prior to the development of contractile dysfunction and heart failure [9]. The causal relationship between PGC1α downregulation and cardiac dysfunction is supported by our data demonstrating that maintenance of PGC1α expression in cardiomyocytes protected against TWEAK-induced cardiac dysfunction. Notably, high degree of chronic overexpression of PGC1α has been associated with the development of cardiac dysfunction [18] and therefore, adenoviral gene expression approaches used in this study aimed to maintain PGC1α in TWEAK-treated cells at control cell levels.

We have previously found that TWEAK-induced cardiac dysfunction requires expression of Fn14 [9]. Fn14 has a short cytoplasmic tail that contains a TRAF consensus-binding motif [28]. The recruitment of TRAFs to the TRAF binding motif leads to activation of signaling cascades and regulates a variety of cellular functions including survival and death, among other functions [29]. To date, there are six known structurally related members in TRAF family, of which TRAF1, TRAF2, TRAF3 and TRAF5 are able to bind with Fn14 [22]. In adult cardiomyocyte, our data have identified TRAF2 as the most abundant TRAF member. TWEAK stimulation was further found to be capable of initiating membrane translocation of TRAF2 in an Fn14-dependent manner. Interestingly, knockdown of TRAF2 with shRNA is sufficient to prevent TWEAK-induced downregulation of PGC1α expression, indicating that TRAF2 is the principal proximal signaling member involved in this event.

A universal characteristic of TWEAK signaling through Fn14 is the activation of NFκB signaling pathway with modulation of numerous downstream target genes [30], [31], [32]. NFκB p65 is present in the cytoplasm and is associated with the repressor protein IκBα. Proinflammatory stimuli such as interleukin-1, TNF, and lipopolysaccharides can activate IκB kinase-β that induces IκBα phosphorylation and proteosome-mediated degradation, which results in NFκB phosphorylation, nuclear translocation, and binding to DNA [33]. In this study, we demonstrate that TWEAK induces IκBα phosphorylation accompanied by IκBα degradation and resynthesis, as well as NFκB p65 phosphorylation in cardiomyocytes, consistent with previous reports [31], [34]. Interestingly, a selective IκB kinase-β inhibitor, SC-514, prevents the downregulation of PGC1α by TWEAK, indicating that TWEAK repression of PGC1α expression requires activation of NFκB signaling pathway. This is consistent with the observation that other cytokines, such as TNFα, suppress PGC1α expression through activation of NFκB [35]. It has also been reported that lipopolysaccharide suppresses PGC-1α gene expression at the transcriptional level, and blocking the toll-like receptor-4/NFκB axis prevented the downregulation of PGC-1 coactivator expression by LPS. However, how activation of NFκB suppresses PGC1α gene expression is unclear and remains under investigation.

In summary, our data suggest that Fn14-TRAF2-NFκB-dependent suppression of PGC1α expression plays a crucial role in TWEAK-induced cardiac dysfunction. Modulation of PGC1α expression or antagonism of Fn14-TRAF2-NFκB may serve as candidate therapeutic targets in preventing TWEAK-induced heart failure.
